# Clinical characteristics and prognosis in patients with urosepsis from intensive care unit in Shanghai, China: a retrospective bi-centre study

**DOI:** 10.1186/s12871-021-01520-5

**Published:** 2021-11-27

**Authors:** Ying Sheng, Wen-long Zheng, Qi-fang Shi, Bing-yu Zhang, Guang-yao Yang

**Affiliations:** 1 Department of Emergency and Critical Care Medicine, Shanghai Pudong Hospital, Fudan University Pudong Medical Center, Shanghai, China; 2 Department of Laboratory Medicine, Shanghai Pudong Hospital, Fudan University Pudong Medical Center, Shanghai, China; 3 Department of Critical Care Medicine, Gongli Hospital Affiliated to Naval Medical University, Shanghai, China

**Keywords:** Urosepsis, Urinary tract infections, Clinical characteristics, Prognosis

## Abstract

**Background:**

The purpose of this study was to retrospectively analyze clinical characteristics and prognostic risk factors of urosepsis patients admitted to two intensive care units in Shanghai, China.

**Methods:**

Clinical data from patients diagnosed with urosepsis were retrospectively retrieved and analyzed from ICU in two regional medical centers from January 2015 to December 2019.

**Results:**

Two hundred two patients were included in the subsequent analysis eventually, with an average age of 72.02 ± 9.66 years, 79.21% of the patients were female and the mortality rate of 15.84%.The proportion of patients with chronic underlying diseases such as diabetes and hypertension was relatively high (56.44, 49.50%, respectively), and the incidence of shock was also high (41.58%) correspondingly. The most common pathogen isolated was *Escherichia coli* (79.20%), of which the extended-spectrum*β*-lactamases (ESBLs)(+) accounted for 42.57%. In multivariate analysis, the strongest predictors for death were mechanical ventilation (OR 7.260, 95% CI 2.200–23.963; *P* = 0.001),chronic kidney disease (CKD) (OR 5.140, 95% CI 1.596–16.550; *P* = 0.006), APACHE II score (OR 1.321, 95% CI 1.184–1.473; *P* < 0.001) and lactate (OR 1.258, 95% CI 1.037–1.527; *P* = 0.020). Both APACHE II score and lactate had the ideal predictive value, with the area under the ROC curve (AUC) of 0.858 and 0.805 respectively.

**Conclusion:**

The patients with urosepsis were characterized by a higher proportion of female, older age, more percentage of comorbidities in this region, and patients with ESBLs (+) *Escherichia coli* infection were more prone to shock. Mechanical ventilation, comorbidity with CKD, APACHE II score and lactate were independent risk factors for death in urosepsis patient, but lactate level and APACHE II score had better predictive value for prognosis.

## Background

Sepsis is one of the most common causes of death among hospitalized patients in intensive care units (ICU) [1]. A recent study showed there were 48.9 million sepsis cases and 11 million sepsis-related deaths worldwide in 2017, making sepsis a global public health problem [2, 3]. Two retrospective studies from China showed that the sepsis mortality exceeded 30% by 2020 [4, 5], making it a heavy burden on the health care system in China.

Sepsis is life-threatening and associated with physiological, pathological and biological abnormalities caused by a dysregulated host response to infections [6]. The common sites of sepsis infection include the lungs, urinary system, abdominal cavity, skin and soft tissue. The different sites of infection may be closely related to the prognosis of patients [7]. Current literature reports show that there are certain differences in the incidence of urosepsis in different countries and regions [5, 8, 9], which may be related to inconsistent diagnostic criteria of urosepsis in recent years.

Even though some studies have summarized the epidemiological characteristics, etiology and prognostic risk factors of urosepsis patients, the susceptibility factors and etiology of patients with urosepsis are different worldwide [10–12]. At present, there are few studies focusing on the prognosis of patients with urosepsis in the ICU, especially no reports from Shanghai, China. The purpose of this study was to retrospectively analyze clinical data and predict the potential risk factors for urosepsis patients admitted to ICUs of two regional medical centers in Shanghai over the past 5 years to help clinicians improve their understanding and clinical prognosis, ultimately contributing to the clinical treatment of patients with urinary system infections.

## Methods

### Study design

A retrospective study was performed in two regional medical centers of Shanghai. Patients with clinical and microbiological diagnoses of septic urinary tract infections admitted to the Department of Critical Care Medicine at Pudong Hospital and Gongli Hospital from January 2015 to December 2019 were enrolled. All patients received standard treatment for sepsis, including antibiotics and fluid resuscitation, as well as mechanical ventilation, vasoactive drugs, and renal replacement therapy, which may be required. The indications of operation were determined by the urologist. They were all monitored in the ICU until their condition improved and they were transferred out of the ICU or died. The study were approved by the Ethics Committee of Shanghai Pudong Hospital of Fudan University and Ethics Committee of Gongli Hospital Affiliated to Naval Medical University, and complied with the Principles of the Declaration of Helsinki. As this study was a retrospective study and medical intervention was not required, the informed consent for this study was waived by the Ethics Committee of Shanghai Pudong Hospital of Fudan University and Ethics Committee of Gongli Hospital Affiliated to Naval Medical University.

### Inclusion and exclusion criteria

Inclusion criteria included the following:1) sepsis caused by a urinary tract infection; 2) Sepsis diagnosed according to the Third International Consensus Definitions for Sepsis and Septic Shock (Sepsis 3).

Exclusion criteria included the following: 1) Concurrent infection at other sites; 2) Malignant tumor patients; 3) Uremia patients undergoing dialysis; 4) Incomplete clinical data.

### Data collection

Data were collected from electronic medical records of two hospitals. Clinical retrospective data was retrieved including age, gender, primary diseases in the urinary system, underlying diseases, comorbidities, laboratory tests, interventions such as the use of mechanical ventilation and Continuous renal replacement therapy (CRRT), etiological results, length of stay (LOS) in ICU and hospital, and hospital mortality. Acute Physiology and Chronic Health Evaluation (APACHE) II and Sequential Organ Failure Assessment (SOFA) scores were also assessed. These clinical data were at their worst condition state within the first 24 h after diagnosis of urosepsis.

### Definitions

Urosepsis is defined as sepsis caused by a urinary tract infection. Sepsis was diagnosed according to the Third International Consensus Definitions for Sepsis and Septic Shock (Sepsis 3) [6] :Sepsis should be defined as life-threatening organ dysfunction caused by a dysregulated host response to infection. For clinical operationalization, organ dysfunction can be represented by an increase in the sepsis-related SOFA score of 2 points or more. Septic shock is defined as persistence of hypotension after adequate fluid resuscitation in patients with sepsis, requiring a vasopressor to maintain a mean arterial pressure of 65 mmHg or greater and serum lactate level greater than 2 mmol/L (> 18 mg/dL).

### Statistical analysis

The SPSS 23.0 statistical software package (IBM, Chicago, IL, USA) was used for statistical analysis analysis. The continuous variables were expressed as mean standard deviation and analyzed using a t-test. The categorical variables were expressed as numbers (percentage) and compared with a chi-square test or Fischer exact test. Variables with a *P*-value < 0.1 in univariate logistic regression analysis were included in multivariate logistic regression analysis. The backward selection method was used to determine the variables included in the final model. Results were expressed as odds ratios (OR) and 95% confidence intervals (95% CI). The discriminatory power of risk factors was determined from the area under the receiver operating characteristic (AUROC) curve with corresponding 95% CIs. Two-sided *P*-values< 0.05 were considered as statistically significant.

## Results

### Demographic and clinical data

**Fig. 1:**
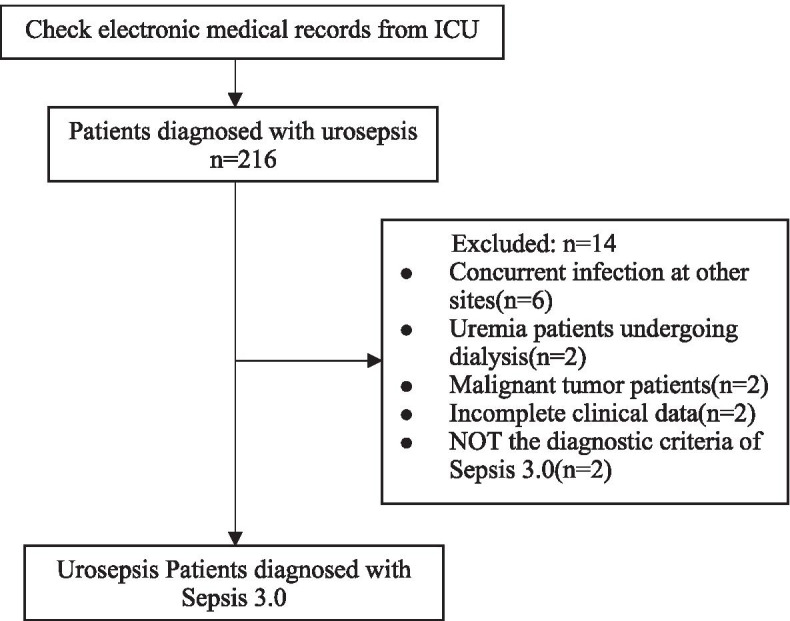
The flow diagram description for inclusion in the study population

A total of 216 hospitalized patients diagnosed with urosepsis were retrieved from the electronic medical records, 14 of whom were excluded from the study and 202 patients were included in the subsequent analysis eventually (Fig. 1). The average age of the enrolled 202 cases was 72.02 ± 9.66 years of age (28–90 years of age), the percentage of patients that were over ≥65 years of age was 76.24% (154/202). A total of 79.21% of the patients were female and a total of 32 of the 202 patients died within 28 days, with the mortality rate being 15.84%. The top two comorbidities observed in these patients were diabetes (56.44%) and hypertension (49.50%). All patients were divided into a survival group and death group. The demographic and clinical characteristics of these patients were summarized in Table 1. There were no differences in age, gender, surgical treatment, comorbidities, long-term indwelling catheter history, white blood count (WBC), hemoglobin (Hb), platelets, C-reactive protein (CRP) and total bilirubin (TBiL) D-dimer between the two groups. The rate of shock in the survival group was significantly lower than in the death group (30.59% vs 62.50%, *P* = 0.001). The APACHE II and SOFA scores were also significantly lower than in the death group (16.75 ± 4.18 vs 24.00 ± 5.59, 5.49 ± 3.36 vs 10.22 ± 3.37; all *P* < 0.001). The proportion of comorbidities with chronic kidney disease in the survival group was 16.47%, which was far lower than the 43.75% observed in the death group (*P* = 0.003). In terms of laboratory tests, WBC, procalcitonin (PCT), Serum creatinine (SCr) and lactate in the survival group were lower than in the death group (all *P* < 0.05), while Hb, albumin and oxygenation index were significantly higher than in the death group (all *P* < 0.05). Within 24 h of ICU admission, the death group needed more mechanical ventilation (*P* = 0.002), but there was no difference in the proportion of CRRT between two groups(*P* = 0.279). Compared with the death group, the survival group had longer hospital LOS (*P* < 0.001), but the ICU LOS was similar.

**Table 1:** Demographic and clinical data of the patients included in this study

	Total (*n* = 202)	Survival group (*n* = 170)	Death group (*n* = 32)	*χ*^*2*^*/t*	*P*-value
Age (years)	72.02 ± 9.66	71.27 ± 9.68	76.00 ± 8.61	2.577	0.011
Female (%)	160 (79.21)	138 (81.18)	22 (68.75)	2.525	0.112
Shock(%)	74 (36.63)	52 (30.59)	20 (62.50)	11.956	0.001
Surgical treatment(%)	170 (84.16)	146 (85.88)	24 (75.00)	2.392	0.122
APACHE II score	17.90 ± 5.16	16.75 ± 4.18	24.00 ± 5.59	8.487	< 0.001
SOFA score	6.24 ± 3.78	5.49 ± 3.36	10.22 ± 3.37	7.284	< 0.001
*Comorbidities*					
Diabetes(%)	114 (56.44)	92 (54.12)	22 (68.75)	2.345	0.126
Hypertension(%)	100 (49.50)	82 (48.24)	18 (56.25)	0.692	0.405
COPD(%)	20 (9.90)	18 (10.59)	2 (6.25)	0.568	0.451
Chronic heart disease(%)	58 (28.71)	52 (30.59)	6 (18.75)	1.844	0.174
Chronic kidney disease(%)	42 (20.79)	28 (16.47)	14 (43.75)	12.169	< 0.001
Cerebrovascular disease(%)	52 (25.74)	40 (23.53)	12 (37.50)	2.750	0.097
Long-term indwelling catheter (%)	26 (12.87)	20 (11.76)	6 (18.75)	1.172	0.279
*Laboratory findings*					
WBC(× 10^9^/L)	17.52 ± 8.79	16.94 ± 8.73	20.58 ± 8.60	2.170	0.031
Hb(g/L)	107.49 ± 13.09	108.33 + 12.70	103.06 ± 14.43	2.107	0.036
Platelet(×10^9^/L)	140.55 ± 69.36	144.72 ± 67.92	118.38 ± 73.78	1.985	0.048
CRP (mg/L)	107.85 ± 61.32	107.25 ± 62.68	111.05 ± 54.32	0.321	0.748
PCT (ng/mL)	49.17 ± 58.05	43.85 ± 53.72	77.43 ± 71.67	3.065	0.002
SCr (μmol/L)	165.90 ± 148.94	148.86 ± 140.46	256.44 ± 161.96	3.877	< 0.001
TBil (μmol/L)	12.64 ± 10.13	12.10 ± 8.14	15.47 ± 17.14	1.734	0.084
Albumin (g/L)	30.25 ± 3.08	30.56 ± 2.92	28.60 ± 3.41	3.388	0.001
D-dimer (mg/L)	6.36 ± 6.80	6.20 ± 7.23	7.25 ± 3.80	0.799	0.425
Oxygenation index (mmHg)	274.39 ± 79.71	281.87 ± 77.96	234.63 ± 78.25	3.143	0.002
Lactate (mmol/L)	3.66 ± 2.33	3.25 ± 1.98	5.83 ± 2.84	6.294	< 0.001
Mechanical ventilation(%)	37 (18.3)	25 (14.71)	12 (37.50)	9.352	0.002
CRRT(%)	26 (12.87)	20 (11.76)	6 (18.75)	1.172	0.279
ICU LOS (days)	9.07 ± 2.66	9.17 ± 2.76	8.56 ± 2.00	1.176	0.241
Hospital LOS (days)	13.14 ± 4.40	13.77 ± 4.41	9.84 ± 2.44	4.882	< 0.001

*APACHE II* Acute physiology and chronic health evaluation II, *SOFA* Sequential organ failure assessment, *COPD* Chronic obstructive pulmonary disorder, *WBC* White blood count, *Hb* Hemoglobin, *CRP* C-reactive protein, *PCT* Procalcitonin, *SCr* Serum creatinine, *TBiL* Total bilirubin; *CRRT* Continuous renal replacement therapy, *LOS* length of stay

### Risk factors and predictive value for prognosis

**Fig. 2:**
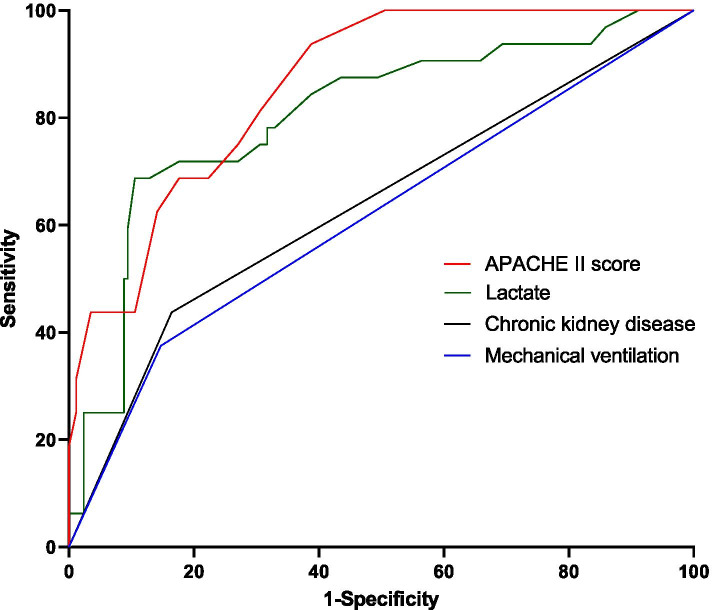
ROC curve analysis of prediction for prognosis in patients with urosepsis. *ROC* receiver operating characteristic, *APACHE II* acute physiology and chronic health evaluation II

To analyze the risk factors associated with death in urosepsis patients with, univariate analysis was performed for thirteen variables, including age, shock, APACHE II score, SOFA score, comorbidity of chronic kidney disease and mechanical ventilation, laboratory tests such as WBC, Hb, PCT, SCr, Albumin, oxygenation index and lactate. Then, multivariate logistic regression analysis was performed for statistically significant results. The data analysis showed that the strongest predictors for death in urosepsis patients were mechanical ventilation (OR 7.260, 95% CI 2.200–23.963; *P* = 0.001), chronic kidney disease (CKD) (OR 5.140, 95% CI 1.596–16.550; *P* = 0.006), APACHE II score (OR 1.321, 95% CI 1.184–1.473; *P* < 0.001) and lactate (OR 1.258, 95% CI 1.037–1.527; *P* = 0.020) (Tables 2 and 3). Then predictive value of these three variables in predicting mortality risk of urosepsis patients was analyzed using the ROC curve. The results showed that both the APACHE II score and lactate had the ideal predictive value, with the area under the ROC curve (AUC) of 0.858 and 0.805 respectively, while comorbidity of chronic kidney disease and mechanical ventilation was 0.636 and 0.614(Table 4, Fig. 2).

**Table 2:** Univariate analysis of variables associated with urosepsis prognosis

	OR	95% CI	*P*-value
Age	1.058	1.012–1.107	0.013
Shock	3.782	1.722–8.304	0.001
APACHE II score	1.307	1.196–1.428	< 0.001
SOFA score	1.398	1.243–1.572	< 0.001
Chronic kidney disease(%)	3.944	1.759–8.845	0.001
WBC	1.046	1.003–1.091	0.034
Hb	0.969	0.940–0998	0.039
PCT (ng/mL)	1.008	1.003–1.014	0.004
SCr (μmol/L)	1.004	1.001–1.006	0.003
Albumin (g/L)	0.805	0.704–0.919	0.001
Oxygenation index (mmHg)	0.991	0.985–0.997	0.003
Lactate (mmol/L)	1.501	1.268–1.777	< 0.001
Mechanical ventilation	3.480	1.514–7.997	0.003

*CI* Confidence interval, *APACHE* *II* Acute physiology and chronic health evaluation II, *SOFA* Sequential organ failure assessment, *WBC* White blood count, *PCT* Procalcitonin, *SCr* Serum creatinine

**Table 3:** Multivariate analysis of variables associated with urosepsis prognosis

	OR	95% CI	*P*-value
Mechanical ventilation	7.260	2.200–23.963	0.001
APACHE II score	1.321	1.184–1.473	< 0.001
Chronic kidney disease	5.140	1.596–16.550	0.006
Lactate	1.258	1.037–1.527	0.020

*CI* Confidence interval, *APACHE* *II* Acute physiology and chronic health evaluation II

**Table 4:** ROC curve analysis of prediction for prognosis in patients with urosepsis

Variables	AUC	95% CI	*P*-value
Mechanical ventilation	0.614	0.500–0.728	0.041
APACHE II score	0.858	0.799–0.918	0.000
Chronic kidney disease	0.636	0.523–0.750	0.014
Lactate	0.805	0.717–0.892	0.000

*CI* Confidence interval, *APACHE* *II* Acute physiology and chronic health evaluation II

### Distribution of pathogens

Pathogenic bacteria were derived from blood and urine cultures of urosepsis patients (Table 5) and a total of 202 strains were identified. Among these strains, 186 (92.08%) were gram-negative bacilli, 4 were (1.98%) gram-positive cocci and 12 (5.94%) were fungi. The most common species of Gram-negative bacteria were *Escherichia coli* (160 strains, 79.20%), of which extended-spectrum*β*-lactamases (ESBLs)(+) accounted for 42.57% (86 strains), ESBLs(−) accounted for 36.63% (74 strains), and others included *Klebsiella pneumoniae* (10 strains, 4.95%), *Proteus mirabilis* (8 strains, 3.96%), *Proteus Penthes* (4 strains, 1.98%), *Klebsiella aerogenes* (2 strain, 0.99%) and *Pseudomonas putida* (2 strain, 0.99%). We compared all pathogen groups and their associated with shock. There were a total of 118 cases and 84 cases in the non-shock group and the shock group, respectively. The results showed that the proportion of *Escherichia coli* ESBLs (+) and *Klebsiella pneumoniae* isolated in the shock group were significantly higher than in the non-shock group (57.14% vs 32.20%, *P*<0.001; 9.52% vs 1.69%, *P* = 0.041), the proportion of *Escherichia coli* ESBLs(−) was obviously lower in the shock versus non-shock group (11.90% vs 54.24%, *P*<0.001), and other groups comparisons were not statistically significant (all *P*>0.05) (Table 5).

**Table 5:** Distribution of pathogens isolated from urosepsis patients experiencing uroseptic shock

	Total (*n* = 202)	Urosepsis (*n* = 118)	Uroseptic shock(*n* = 84)	*χ*^*2*^	*P*-value
**Gram-negative bacteria(%)**	186 (92.08)	112 (94.92)	74 (88.10)	3.129	0.077
*Escherichia coli* ESBLs(−)	74 (36.63)	64 (54.24%)	10 (11.90%)	37.881	<0.001
*Escherichia coli* ESBLs(+)	86 (42.57)	38 (32.20%)	48 (57.14%)	12.483	<0.001
*Klebsiella pneumoniae*	10 (4.95)	2 (1.69%)	8 (9.52%)	4.186	0.041
*Proteus mirabilis*	8 (3.96)	4 (3.39%)	4 (4.76%)	0.016	0.889
*Proteus penneri*	4 (1.98)	2 (1.69%)	2 (2.38%)	0.000	1.000
*Klebsiella aerogenes*	2 (0.99)	0 (0.00%)	2 (2.38%)	NA	NA
*Pseudomonas putida*	2 (0.99)	2 (1.69%)	0 (0.00%)	NA	NA
**Gram-positive bacteria(%)**	4 (1.98)	2 (1.69%)	2 (2.38%)	0.000	1.000
*Staphylococcus aureus*	4 (1.98)	2 (1.69%)	2 (2.38%)	0.000	1.000
**Fungus(%)**	12 (5.94)	4 (3.39%)	8 (9.52%)	2.298	0.130
*Candida albicans*	8 (3.96)	2 (1.69%)	6 (7.14%)	2.531	0.112
*Candida pseudotropicalis*	2 (0.99)	0 (0.00%)	2 (2.38%)	NA	NA
*Candida glabrata*	2 (0.99)	2 (1.69%)	0 (0.00%)	NA	NA

*ESBLs* Extended-spectrum*β*-lactamases

## Discussion

In the current study, a retrospective analysis was performed using clinical data of urosepsis patients admitted to two ICUs over the past 5 years using sepsis 3.0 diagnostic criterion and this was also the first clinical analysis performed in this region to our knowledge. Our study indicated that the majority of urosepsis patients admitted to the ICU were elderly and nearly 80% of the patients were women. The age and proportion of women were obviously higher than what were observed in the study reported by Xin-Hua Qiang et al. [11], who found the average age to be 43.6 ± 12.5 years old and the percentage of females to be 53%. A study performed by Ying Jiang et al. [10] reported that the average age of urosepsis patients was 59.85 years old and 50% were women from Mainland China. In addition, the mortality rate was found to be 15.84% in this study. A possible explanation for this high mortality rate may be related to the fact that their hospitals are large provincial and municipal medical centers [10, 11], while our hospital is a regional medical center. The patients in this study were older, with a higher proportion of patients having chronic underlying diseases, such as diabetes and hypertension and there was also a greater incidence of shock. A study from a regional medical center in Taiwan showed that compared with patients younger than 65, elderly urosepsis patients over the age of 80 have a significantly higher risk for shock (OR 1.99, *P* = 0.004), but men show an increased risk of shock (OR 1.54, *P* = 0.022 )[12]. In addition, our study revealed that 12.87% of patients showed a history of long-term indwelling catheterization, which may be related to up to 25.74% of patients with previous cerebrovascular disease. While the length of stay in the ICU was similar in both groups, the length of hospital stay in the survival group was longer, which is similar to the results of a multicenter study from mainland China [5].

Multivariate logistic regression analysis revealed that the OR of chronic kidney disease was 5.140, suggesting that urosepsis patients complicated with chronic kidney disease have a high risk of death. This was also supported by a study performed by Yi-wenn Yvonne Huang et al [13]. Many epidemiological studies revealed significant associations between chronic kidney disease with poor outcomes in sepsis patients that may be related to the accumulation of inflammatory cytokines attenuating immune function caused by decreased renal clearance, platelet dysfunction and thrombocytopenia in patients with chronic kidney disease [14]. Even though our analysis showed that the proportion of diabetes and hypertension in comorbidities was higher and the ratio of chronic heart failure reached 28.71%, none showed an increase in the risk of death. This result differs than what was reported by Sinapidis D et al. [15] and Chen PY et al. [16], which may be associated with the demographic characteristics of this study population. As the subjects of this study were urosepsis patients, the proportion of patients requiring mechanical ventilation was not high. Our study showed that the OR of mechanical ventilation was 7.260, indicating that these patients had more severe systemic organ function damage, leading to a worse prognosis.

Hyperlactatemia (usually ≥4 mmol/L) represents low perfusion in sepsis patients tissues, which has been included in the 2016 SSC guidelines for the early identification of sepsis and septic shock [6], also closely correlated with worse prognosis of sepsis patients [17]. Although some evidence has suggested that the APACHE II score may provide inaccurate information in the certain patients, such as unconscious patients may score too high, however, it is undeniable that it is still the most widely used score to evaluate critically ill patients so far, and it has a good prognostic evaluation value for patients with sepsis [18, 19]. Our results revealed that compared with mechanical ventilation and chronic kidney disease, both the lactate level and the APACHE II score had better predictive value for the prognosis of patients with urosepsis, which indicated that lactate level and the APACHE II score may provide a better risk assessment and may be useful as a prognostic marker for urosepsis patients.

In urosepsis, the most commonly isolated pathogen is *Escherichia coli*, followed by other types of *Enterobacteriaceae*. In contrast to pulmonary or abdominal sepsis, most urosepsis cases are caused by a single microorganism [20]. Our retrospective analysis showed that the vast majority of isolated pathogens were gram-negative bacteria. *Escherichia coli* accounted for 86.02% of all gram-negative bacteria and 79.2% of all bacteria, both were significantly higher than the 75 and 58%, respectively reported by Xin-Hua Qiang et al. [11], and the 64.62 and 48.28%, respectively reported by Ying Jiang et al. [10]. In addition, our data showed that more than half of the 160 *Escherichia coli* strains isolated were ESBLs (+). Such a high resistance trend is similar to the results from a multicenter study performed in Europe in 2015 [21]. A study from Canada showed that in 176 *Escherichia coli* strains isolated from blood cultures of urosepsis patients, the ratio of ESBLs (+) and ESBLs (−) was 1:2 [13]. In a study performed by Ying Jiang et al. [10], 34 of the 42 *Escherichia coli* strains were ESBLs (+), and the ratio of ESBL (+) and ESBL (−) reached 4:1. Even though some of these studies contained small sample sizes, they still indicated that different geographical and demographic differences, economic differences and even prescription behavior may lead to different distributions of drug-resistant bacteria, thus affecting prognosis [11, 22–24]. In addition, subgroup analysis showed that the proportion of ESBLs (+) of *Escherichia coli* in the uroseptic shock group was significantly higher than that the non-shock group(*P*<0.001).

Besides, this study further showed the proportion of *Enterobacteriaceae* was 90.10%, which was significantly higher than what was reported by Xin-Hua Qiang et al. [11] (66.67%) and Ying Jiang et al. [10] (67.82%), and also higher than what was reported by the European multicenter study performed in 2015 [21], showing that *Enterobacteriaceae* accounted for 43% of all the isolated pathogens. This is beneficial for the early and rapid anti-infection treatment of patients with sepsis to regularly monitor pathogenic distribution and drug resistance mechanisms of common infections in the region. For the initial antibiotic treatment of urosepsis patients in areas that have high incidence of ESBLs (+), the generally recommended antibiotics are carbapenems, but clinicians need to be alert to the production of *carbapenemase* strains [25].

Upper urinary tract obstruction is an important cause of urosepsis and is the most common cause of urinary tract stones. Surgical interventions such as shock wave lithotripsy and percutaneous nephrolithotripsy (PCNL) are the main treatments of upper urinary tract stones [26]. PCNL may cause excessive pressure in the renal collecting system during an operation and a large number of bacteria or toxins will enter the blood and thus lead to sepsis [27]. Our study also found that up to 84.16% of patients had a history of surgery. Unfortunately, since this was a retrospective study, we were still unable to determine which patients were secondary to urogenic sepsis after surgery. The above issues need to be further analyzed and confirmed by future research.

The treatment of urosepsis requires interdisciplinary, including emergency unit, urological specialties, and intensive care medicine [28, 29]. At present, most clinical studies of urosepsis are written from the perspective of urologists, but there are few clinical research papers conducted from the perspective of ICU physicians. The advantage of this study is that we have explored the clinical characteristics and prognostic factors of patients with urosepsis in our region from the perspective of the ICU to identify patients with high risk of death and provide early intervention. To our knowledge, this is the first retrospective study of urosepsis patients in ICU in Shanghai, China, with the larger sample size compared with other clinical studies to date. However, this study still faces some limitations that should be pointed out. First, as a retrospective study, some unmeasured confounders might influence the final results. But we conducted the study in the 2 large medical centers with adequate sample size to minimize the underlying effects. Second, since most patients had a history of emergency surgeries and patients without surgery were also admitted to ICU from the emergency department, community-acquired infections may have contributed to the differences observed in our study versus some others. Third, a small number of patients may be associated with the spread of bacterial toxins during surgery, but we cannot accurately distinguish these patients. We plan to conduct prospective studies involving more large regional medical centers to further confirm these findings.

## Conclusion

The patients with urosepsis were characterized by a higher proportion of female, older age, and more percentage of comorbidities in this region. *Escherichia coli* was more common in etiology, and patients with ESBLs (+) *Escherichia coli* were more prone to shock. Mechanical ventilation, comorbidity with chronic kidney disease, APACHE II and lactate were independent risk factors for death in urosepsis patient. Lactate level and the APACHE II score had better predictive value for prognosis.

## Data Availability

In order to protect patient privacy, the datasets used and analyzed are not publicly available, but they are available from the corresponding author on reasonable request.

## Abbreviations

ICU: Intensive care units
PCNL: Percutaneous nephrolithotripsy
APACHE II: Acute physiology and chronic health evaluation II
SOFA: Sequential organ failure assessment
COPD: Chronic obstructive pulmonary disorder
WBC: White blood count
Hb: Hemoglobin
CRP: C-reactive protein
PCT: Procalcitonin
SCr: Serum creatinine
TBiL: Total bilirubin
ESBLs: Extended-spectrum*β*-lactamases
